# An App for Care Partners of Persons Living With Dementia (Aliviado Caregiving): Human-Centered Design Study

**DOI:** 10.2196/82862

**Published:** 2026-08-27

**Authors:** Moroni Fernandez Cajavilca, Shih-Yin Lin, Aditi Durga, Sasha Perez-Greenemeier, Kimberly Cheng Hom, Denise Lawson, Abraham Brody

**Affiliations:** 1 College of Nursing New York University New York, NY United States; 2 Brookdale Department of Geriatrics and Palliative Medicine Icahn School of Medicine at Mount Sinai New York, NY United States; 3 Pop Services White Plains, NY United States

**Keywords:** caregiving, care partner, co-design, digital health, mobile app

## Abstract

**Background:**

Behavioral and psychological symptoms of dementia (BPSD) are among the most challenging aspects of dementia care and contribute substantially to care partner burden. Family care partners, who provide most community-based dementia care, often lack access to evidence-based, nonpharmacological interventions for managing BPSD, particularly in underserved communities. Mobile health (mHealth) apps offer a promising avenue to expand care partners’ access to caregiving support. However, existing dementia caregiving apps frequently provide generalized information rather than personalized guidance based on care partners’ priorities.

**Objective:**

This manuscript describes the iterative, human-centered design process used to develop and tailor the Aliviado Caregiving mHealth app, designed to support care partners of persons living with dementia in prioritizing and managing BPSD through evidence-based, nonpharmacological strategies.

**Methods:**

Guided by an iterative review and revision framework and a coproduction innovation pathway, the development of the Aliviado Caregiving app occurred across 3 stages. Key participant groups were recruited across these stages, including an investigator team and clinician users (n=101) in stage 1, care partners of persons living with dementia (n=8) in stage 2, and a community advisory board (n=9) in stage 3. Quantitative data from stage 1 were analyzed using descriptive statistics to summarize clinician characteristics and identify top clinician-recommended features for care partners’ BPSD self-management. Qualitative data in stages 2 and 3 were analyzed through conventional content analysis.

**Results:**

Clinician survey findings revealed that the top app features to support family care partners’ self-management of BPSD included “caregiver stress management strategies/intervention” (85/101, 84%), “dementia symptom management education and resources” (74/101, 73%), and “caregiver stress self-monitoring/tracking” (70/101, 69%). Two overarching qualitative categories were identified: (1) facilitators of care partner engagement and (2) desired features for a dementia caregiving app. Participants across stages emphasized individualized BPSD decision support, tailored terminology, and self-monitoring through reflective journaling to facilitate engagement. Participants also recommended future design enhancements, such as multilingual support, including Spanish-language functionality; support for multiple care recipients and care partners; and continuously updated educational resources to support diverse caregiving needs.

**Conclusions:**

The Aliviado Caregiving app was developed through a rigorous, human-centered design process. Co-design with clinicians, care partners, community advisory board members, and a faith-based leader enhanced the relevance and usability of this mHealth app while supporting sustained engagement. Future research will evaluate the app’s acceptability and feasibility in reducing care partner burden and improving BPSD management in a pilot project.

## Introduction

Care partners such as adult children and spouses provide most care to persons living with dementia living in the community. A nationally representative sample found that care partners of persons living with dementia were more engaged in most types of assistance than care partners of adults without dementia, including household activities (92% vs 87%), health care system interaction (79% vs 61%), health or medical care (76% vs 60%), and self-care or mobility activities (71% vs 60%) [1]. The ability to carry out these activities is significantly impacted by behavioral and psychological symptoms of dementia (BPSD), such as agitation, depression, or wandering, and care partners often receive limited training or support in managing these symptoms, while health care team members may also lack awareness of evidence-based management strategies, particularly nonpharmacologic approaches [2].

BPSD significantly increases care partner burden, often leading to stress, burnout, and poor physical and mental health [3]. The bidirectional relationship between BPSD and care partner well-being means that worsening symptoms in persons living with dementia can intensify care partner strain, which in turn exacerbates the symptoms of dementia [4]. Care partners frequently struggle to determine which BPSD to prioritize, as symptoms vary in severity and impact. For instance, although agitation may pose immediate safety concerns, depressive symptoms could undermine the overall quality of life for both the care partner and the persons living with dementia. These challenges are especially pronounced in underserved communities, where care partners often lack access to any resources or support, let alone ones tailored for their specific communities, compounding the difficulty of managing BPSD effectively [5].

Disparities in support for care partners are significant, particularly among those from marginalized communities. Care partners from these underserved communities often find greater meaning in positive aspects of caregiving, which can have a protective effect, yet they also often lack access to evidence-based training or high-quality nonpharmacological interventions for BPSD [6,7]. Black and Latino care partners report higher care needs, less access to paid care services, and greater levels of depression than non-Hispanic White care partners [8]. In addition, most care partner intervention studies have not even reported a breakdown of race or ethnicity among participants [6]. These disparities highlight the need for accessible, culturally responsive tools to support care partners. Mobile health (mHealth) interventions and care partner–facing apps have emerged as a promising approach to expand access to evidence-based education and support for dementia care partners. However, prior reviews suggest that these apps often rely on static educational materials and generalized resources, with few offering structured BPSD decision support or tailoring content to care partners’ prioritized concerns, time constraints, and contextual needs [9,10]. In addition, reports describing the development and evaluation of these apps often included limited representation of care partners from historically marginalized communities, which can constrain their interpretability and generalizability [11].

Building on prior literature and input from community-based clinical teams and dementia support organizations, we developed the Aliviado Caregiving mHealth app. This approach is built on the original Aliviado Dementia Care model to support clinical teams in BPSD management, which is conceptually guided by the structural model of caregiving stress [12]. The purpose of this study was to describe the iterative, coproduction approach undertaken in the development of the Aliviado Caregiving wireframes, with the intention of improving the app’s feasibility and acceptability for future pilot testing.

## Methods

### Overview

**Figure 1:**
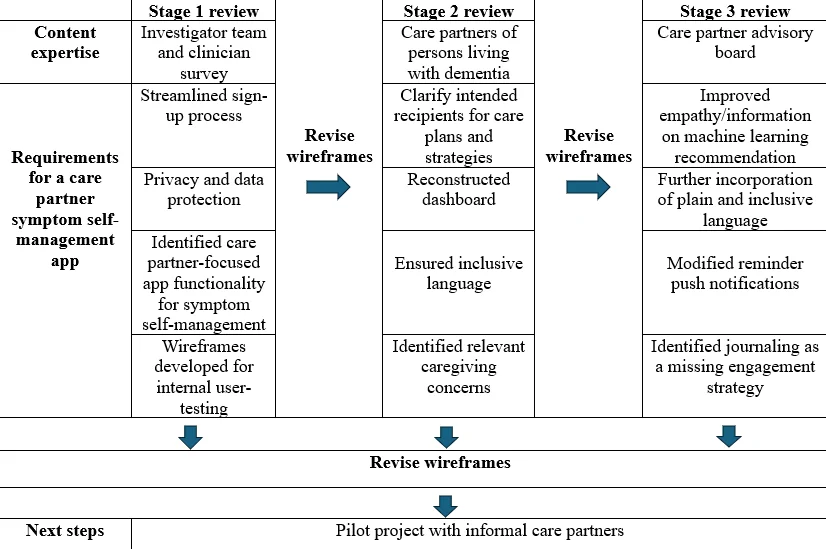
Aliviado Caregiving 3 stage review process and wireframe revisions.

The development of the Aliviado Caregiving app (New York University) involved an iterative 3-stage process to receive and incorporate feedback into the adapted wireframes. These 3 stages were guided by an iterative review and revision template [13] and followed the coproduction approach of the production innovation pathway [14]. Prior to initiation of the formal coproduction process, several hospice clinicians reported challenges in supporting care partners during a pilot phase of a pragmatic trial that incorporated an mHealth app for clinicians but not care partners [15]. Accordingly, in stage 1 of the study, we conducted a needs assessment (innovative ideas phase) that involved both the investigator team and a clinician survey. This was followed by stage 2 (development phase), during which care partners of persons living with dementia engaged in individual think-aloud sessions. In these sessions, updated wireframes were presented, feedback was collected, and additional ideas regarding functionality and user interface design were elicited. We then established a care partner advisory board (CAB) in stage 3, which contributed to planning the coproduction process (planning phase) and participated in generative feature development and wireframe review (continued development phase). We designed and revised the Aliviado Caregiving wireframes between each review stage to address expert feedback (Figure 1).

### Ethical Considerations

All stages were approved by the New York University Grossman School of Medicine Institutional Review Board (IRB). In stages 1 and 2, informed consent was obtained. In stage 3, although included in the approved IRB protocol, this stage was not classified by the IRB as human subjects research. The CAB functioned in an advisory and partnership capacity with the study team, focusing on how to address feedback from stages 1 and 2. As such, stage 3 was considered a quality improvement activity rather than generalizable research and did not require formal informed consent. Nevertheless, the process was explained to participants, and verbal consent was obtained for participation in and recording of CAB meetings. A US $50 incentive gift card was provided upon interview completion for the care partners of persons living with dementia. CAB members received a US $50 gift card for each individual and/or focus group meeting they attended.

### Materials and Procedures

#### Stage 1: Needs Assessment by the Investigator Team and Clinician Survey

The investigator team identified the minimum requirements for a care partner app for BPSD self-management, building on findings from our previous nationwide pragmatic trial of the comprehensive dementia symptom management program, Aliviado Dementia Care, and developed an initial set of wireframes using Figma (Figma Inc) [15]. This trial included a clinician-centered mHealth app as part of the Aliviado Dementia Care program intervention to assist in assessing patients and supporting care partners in BPSD management. During the trial, clinicians at multiple sites remarked on the need for empowering care partners and providing a companion app for them to use to address BPSD. Clinicians also shared that care partners had asked about the clinician app and why they could not have access to it when it was being used in their homes. The investigator team, therefore, surveyed clinicians participating in the pragmatic trial who used the clinician version of the Aliviado symptom management app to prioritize key potential features for the development of a care partner counterpart, later named the Aliviado Caregiving app. The recruitment process, eligibility criteria, and procedures of the clinician survey were published elsewhere, although the results focused on the usability of the Aliviado clinician app and not on the development of the Aliviado Caregiving app [16]. Briefly, a convenience sample of 101 clinician users completed an online Qualtrics (Qualtrics Inc) survey between June and July 2020. In the survey, clinicians were asked which app features they considered most helpful to include in a new care partner version of the BPSD self-management app [16]. On the basis of the features identified by the investigator team and prioritized by clinician users, a set of draft wireframes was developed to support care partner BPSD self-management and to help anchor further discussion, usability testing, and co-design with care partners and the CAB during stages 2 and 3, as detailed in the following sections.

#### Stage 2: Review and Usability Testing With Care Partners of Persons Living With Dementia

We recruited care partners aged ≥18 years who spoke English, possessed a smartphone, and dedicated a minimum of 4 hours a week to caring for a person living with dementia [17]. Our recruitment strategies included registration on the TrialMatch and ClinicalTrials.gov websites in collaboration with the Alzheimer’s Association. Oral consent was obtained, and individual Zoom (Zoom Communications Inc) think-aloud usability testing sessions (approximately 30-45 minutes) were conducted by a trained project manager (AD) using a semistructured guide and screen-shared wireframes between October and December 2022. Participants were asked to speak their minds while viewing and interacting with the wireframes (eg, asking the interviewer to go back and forth between wireframes, clicking on buttons, or enlarging certain elements on the screen) [17]. Example questions or prompts presented to participants included the following: “What are your impression of the signing up process?” “Was this process clear to you?” “Is this what you were expecting to see?” “Do you feel anything is missing?” “What do you think the purpose of this screen is?” and “How might this be of value to you?” Participants were offered an optional brief demographic survey to reduce burden and protect privacy. Therefore, demographic completeness varies, and the interpretability of sample characteristics is limited.

#### Stage 3: Review, Usability Testing, and Co-Design With the CAB

We created a 9-member CAB using purposive, community-based recruitment. We purposively partnered with an ordained clergy member (DL) of an African American church who also runs a caregiving support program to identify potential CAB members who were current or former care partners of persons living with dementia and from historically marginalized communities. CAB members were asked to provide content expertise and lived-experience input on design, features (both to include and remove), usability, language, and recommendations for the care partner–facing workflow. This composition provided valuable lived-experience feedback from a group often underrepresented in digital health design. However, it also limits the transferability of findings to care partner groups not represented in this sample. One focus group (approximately 1 hour) was led by the experienced ordained clergy member (DL) in July 2024 (5 CAB members attended). In addition, individual virtual meetings (approximately 30-45 minutes), following a format similar to that of the stage 2 think-aloud sessions, were conducted in August 2024 with all CAB members by the senior research scientist (SYL), project manager (KCH), and research assistant (MFC), all of whom have qualitative research experience.

### Data Analysis

#### Quantitative Data Analysis

Descriptive statistics, including frequencies and percentages, were calculated using RStudio (Posit Software PBC) to summarize clinician characteristics in stage 1 and rank clinician-recommended key features for display in the initial wireframes in preparation for further iteration, usability testing, and co-design in stages 2 and 3 [16].

#### Qualitative Data Analysis

Qualitative transcripts were analyzed using conventional content analysis [18,19]. Two reviewers (SYL and MFC) independently immersed themselves in the transcripts and conducted inductive coding. Codes and categories were derived directly from the data rather than from predefined theoretical constructs [18]. Preliminary codes and categories were reviewed with the full author team, and categories were iteratively refined through the analytic process [18,19]. To enhance credibility, multiple discussions were held with the full research team to review and validate the analysis, and member checking was incorporated across all 3 stages to support iterative revision and ensure alignment with the perspectives of the investigator team, care partners, and CAB. Representative quotes and participant pseudonyms are included to illustrate key findings. Confirmability was addressed through discussions among the research team to acknowledge and minimize potential researcher bias throughout the analytic process. A COREQ (Consolidated Criteria for Reporting Qualitative Research) checklist was also used to enhance methodological rigor and reporting transparency (Multimedia Appendix 1) [20]. Finally, to enhance transferability, we provide detailed descriptions of the study context.

## Results

### Overview

The goal of the Aliviado Caregiving app is to support care partners of persons living with dementia in prioritizing and managing BPSD using evidence-based, nonpharmacological strategies (Table 1). The app was adapted from the original Aliviado Dementia Care model, which included a clinician-centered mHealth app to support BPSD assessment and management. In the present study, initial care partner–facing wireframes were developed and then revised across 3 stages based on feedback from the investigator team and from clinicians, care partners, and a CAB.

**Table 1:** Evidence-based nonpharmacologic strategies presented to address each behavioral and psychological symptoms of dementia (BPSD) in the Aliviado Caregiving app at the time of analysis.

BPSD	Nonpharmacologic strategies
Agitation	Aromatherapy; music; pet therapy; validate, redirect, and distract
Anxiety	Aromatherapy, art therapy, music, pet therapy, and physical activity
Apathy	Art therapy, music, pet therapy, and social outings and activities
Depression	Aromatherapy, art therapy, good sleep practices, life review, music, pet therapy, physical activity, reminiscence therapy, and social outings and activities
Disinhibition	Good sleep practices; physical activity; strategies for reducing inappropriate sexual behavior; social outings and activities; validation, redirection, and distraction
Hallucinations	Good sleep practices; validation, redirection, and distraction
Motor behaviors	Activity aprons; environmental modifications; music; pet therapy; physical activity; validation, redirection, and distraction
Sleep problems	Good sleep practices, music, and physical activity

### Overview of Participants and Themes

Our iterative review process involved an investigator team and 101 clinician users, 8 care partners, and 9 CAB members. The stage 1 investigator team consisted of professional experts working in the field of dementia care (eg, researchers). Most clinician users were nurses (45/101, 44.8%) and were White (75/101, 74%) [16]. Stage 2 care partners were identified as White (4/8, 50%), Black or African American (3/8, 37.5%), or Asian (1/8, 12.5%) [17]. The care partners were primarily female (5/8, 63%), had completed high school as the highest level of education (5/8, 63%), and were widowed (5/8, 63%) [17]. The stage 3 CAB members included a total of 9 (100%) care partners who were primarily female (n=6, 67%), and all members self-identified as Black or African American (n=9, 100%).

The qualitative findings highlight how prominent suggestions and changes across iterative review stages evolved from clinician-informed design priorities to care partner– and CAB-driven refinements, which coalesced into two main categories: (1) facilitators of care partner engagement and (2) desired features for a caregiving app, each with their respective subcategories.

### Category 1: Facilitators of Care Partner Engagement

This category reflects care partners’ preferences for app engagement strategies that encourage sustained use through the subcategories of user-centered design, personalized terminology, and personalized guidance and strategies, with the overarching goal of reducing burden and enhancing the caregiving experience.

#### Continuity Through Self-Monitoring and Reflection

Care partners and CAB members consistently expressed the importance of app features that support ongoing self-monitoring and reflection as a means of allowing sustained engagement over time. Participants noted that visual indicators of progress, such as daily tracking figures, reinforced continuity in implementing care strategies and allowed them to observe their progress. One care partner expressed the following:

Liked the happy face. It’s good to track [care strategy progress] daily to see how you are getting better.

Care partner 1

In addition, care partners identified journaling as a missing app feature that could further strengthen engagement by providing a dedicated space to document caregiving experiences, reflect on daily challenges, and monitor changes over time. One CAB member highlighted the value of reflective journaling, recommending the following:

A journal, reflection space for caregivers at the end of the day.

CAB 2

#### Personalized Terminology

Care partners stressed that personalized terminology enhanced the relevance and emotional connection of the app, thereby supporting continued engagement. During stage 1, the investigator team incorporated greater flexibility by allowing users to replace generic labels with a chosen relationship term (eg, “father” and “mother”)—a change intended to better honor the personhood of the persons living with dementia and reflect familiar social roles. However, participants identified opportunities for further personalization. During stage 3, 1 CAB member recommended replacing relationship labels with the individual’s preferred name to create a more personal and meaningful user experience:

Instead of assessing your father’s, say the name of the persons living with dementia, becomes more personal.

CAB 7

#### Personalized User-Concern Decision Support

**Figure 2:**
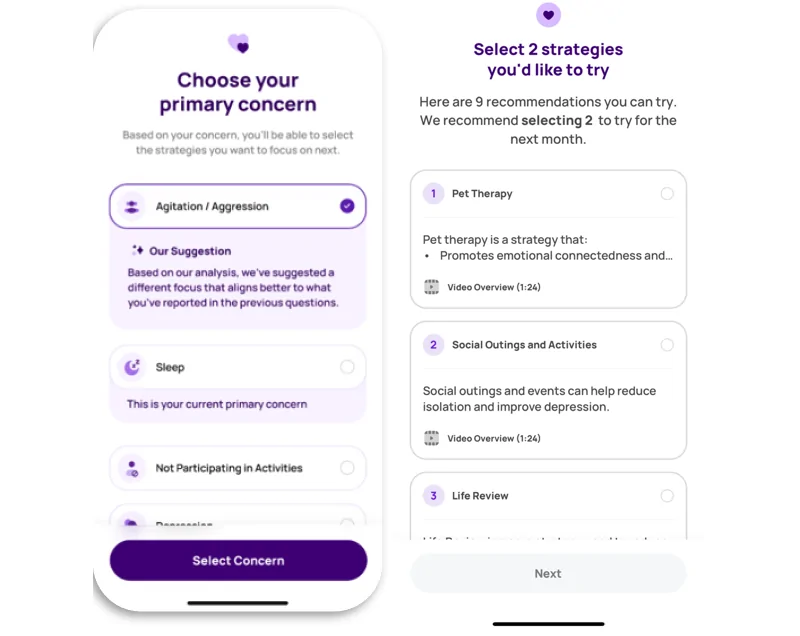
Revised prioritization using an AI/machine learning algorithm and care strategy selection wireframes based on participant feedback.

Care partners valued individualized recommendations that reflected their priorities and unique caregiving contexts. The Aliviado Caregiving app was built to offer a flexible, personalized approach that would allow care partners to prioritize BPSD they perceived as most pressing while receiving evidence-based guidance tailored to their needs. A BPSD prioritization engine was added in stage 1 based on participant feedback to enhance personalization and promote sustained engagement with the app (Figure 2). Care partners were asked to identify a primary BPSD concern and complete a BPSD and associated stress assessment (using the Neuropsychiatric Inventory Questionnaire) within the app. The engine then guided care partners in selecting an evidence-based, nonpharmacological care plan by either choosing the BPSD they initially identified or following an algorithm-generated BPSD management recommendation (decision support) based on the assessment results and the BPSD’s predicted potential to reduce care partner stress if adequately managed.

During stage 2, care partners reported that the prioritization engine increased the app’s relevance by supporting personalized decision-making while providing actionable care recommendations. Participants acknowledged the ability to either prioritize concerns based on their caregiving experiences or accept the app’s recommendations. One care partner noted that it enabled them to receive the following:

A particular plan that better works for your current situation, for both [persons living with dementia and care partner].

Care partner 7

Another participant stated how the prioritization process translated assessment into meaningful action:

Now you can implement it because you have determined something [care strategy], and you have a [care] plan you can implement.

Care partner 2

Feedback from the stage 3 CAB further refined the prioritization engine to better support user engagement and trust. CAB members emphasized that when the app recommended a BPSD that differed from the care partner’s perceived priority, the rationale should be clearly explained to avoid confusion or minimizing their lived experience. One CAB member expressed the following:

I am dealing with this day-to-day...responding to a questionnaire, and do I doubt, or do you doubt what I am saying, based on my experience vs. your analysis [algorithm]. Somewhere along the lines provide an explanation why the recommendation is vastly different...How did you get here from what I told you?

CAB 3

The refinements made between stages strengthened the transparency, flexibility, and user-centered design of the prioritization engine, thus fostering greater trust and continued engagement with the app.

### Category 2: Desired Features for a Dementia Caregiving App

#### Overview

This category represents the functional content and capabilities that the investigator team and care partners identified as essential for supporting dementia caregiving. During stage 1, clinicians prioritized several core features for the care partner app, including “care partner stress management strategies/intervention” (85/101, 84%), “dementia symptom management education and resources” (74/101, 73%), “care partner stress self-monitoring/tracking” (70/101, 69%), and “dementia behavioral symptom monitoring/tracking” (60/101, 59%) [16]. These findings informed discussions among the investigator team and guided the development of the initial care partner app wireframes. Across subsequent stages, care partners further expanded upon these priorities by identifying additional functionalities that would improve the app’s usefulness, organized into 2 subcategories: evidence-based educational content and support and features addressing diverse caregiving needs.

#### Dynamic Evidence-Based Educational Content and Support

Care partners voiced the importance of dementia caregiving apps having access to educational resources that were evidence based and continuously updated. Participants expressed a preference for educational content that extended beyond static information and emphasized the need for resources that reflect current evidence and emerging best practices. Several care partners also noted the value of being able to revisit educational materials over time. One participant stated the following:

Does it update current information?... I would want to bookmark have new information.

Care partner 4

In addition to up-to-date educational materials, care partners wanted resources that translate evidence into actionable caregiving strategies tailored to their immediate concerns. Participants expressed that caregiving apps should not only identify BPSD but also pair those concerns with practical, evidence-based recommendations and implementation guidance. One care partner explained the following:

...pretty prominent issue for me, and you are noting it [care strategy] and offering resources and how to implement dealing with that. You are implementing suggestions and tools to help and reduce the agitation and aggression [BPSD].

CAB 4

#### Features Addressing Diverse Caregiving Needs

Participants envisioned dementia caregiving apps such as Aliviado Caregiving to be adaptable to the diverse circumstances and responsibilities of care partners. They expressed the importance of designing features that accommodate varying linguistic, cultural, and caregiving needs to improve accessibility and usability across different populations. For instance, 1 CAB member underscored the need for a Spanish-language version of the app to better serve the growing population of Spanish-speaking care partners of persons living with dementia. Participants also recognized that caregiving responsibilities may extend to “more than one care recipient” (care partner 1) or that care partners may wish to invite additional family members or professional aides to participate in tracking a single care recipient, and they recommended features that reflect these caregiving realities. Collectively, these findings indicate that care partners value flexible and inclusive app features capable of accommodating the diverse and evolving demands of dementia caregiving.

## Discussion

### Principal Findings

**Figure 3:**
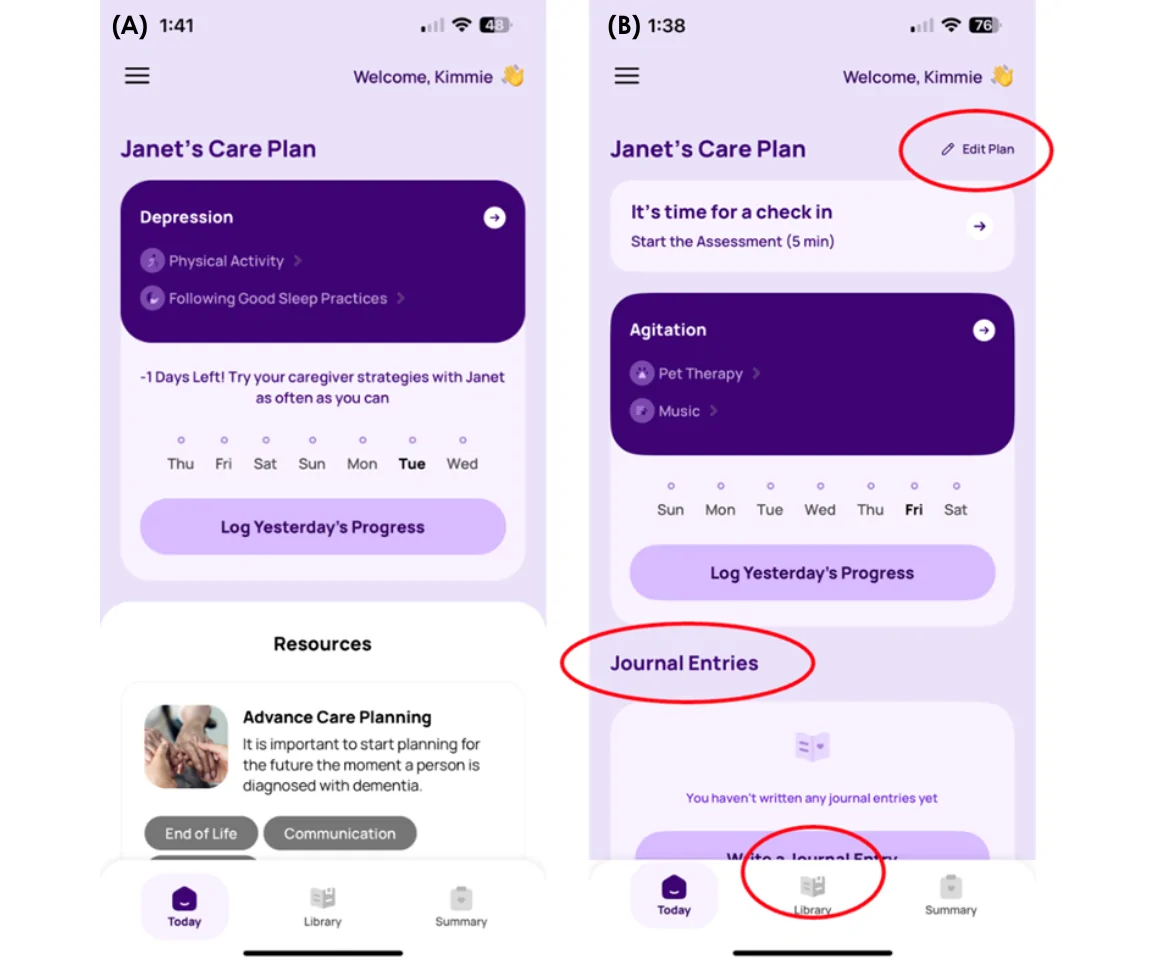
Aliviado Caregiving app home page and selected features: (A) initial version and (B) revised version after iterative feedback.

We used an iterative, human-centered design process to develop the Aliviado Caregiving app, engaging clinicians, care partners, and a CAB across 3 stages. An example of revisions following iterative feedback across all stages is illustrated in Figure 3. The primary contribution of this manuscript is to offer generalizable insights into applying participatory, human-centered design to dementia caregiving apps that build upon existing evidence-based programs whose “active ingredients” (translated into the apps’ minimal requirements) must be retained to ensure intervention effectiveness, all while incorporating recommendations from existing app users (clinicians) and the lived experiences and preferences of new target users (care partners). Specifically, we describe a development process that concurrently translated an interdisciplinary, organizational-level care model into a care partner–centered self-management app and extended a clinician-focused symptom management app to incorporate care partner–directed support. This case illustrates how iterative, community-engaged methods can inform the adaptation of clinician-facing digital mHealth interventions into care partner–focused mHealth tools, while maintaining transparency about scope and transferability and laying the groundwork for subsequent pilot testing to evaluate feasibility and acceptability outcomes. Of note, although we initially had goals for the app’s purpose and some initial wireframes for care partners to react to, we did not move forward with building a functional app until the stage 3 CAB had provided substantial idea generation and feedback, thus allowing significant flexibility and responsiveness to the needs they endorsed.

The greatest strength of this study was addressing common limitations of human-centered design in developing the Aliviado Caregiving app. These typical limitations include sample bias (eg, predominantly White samples), overreliance on early end-user input, and ethical or societal issues that may be unaddressed at the macrolevel when focusing on individual users [21]. First, we attempted to mitigate sampling bias by partnering with local partners and organizations to recruit a diverse sample of care partners representing varied perspectives and caregiving contexts. A key innovation in developing our co-designed app was engaging a faith-based leader to establish and facilitate the CAB in stage 3. Faith-based leaders can serve as trusted community messengers with established relationships and broad reach across diverse community networks, while bringing compassionate and supportive dimensions to health engagement [22,23]. This community-engaged methodological approach was therefore intended to foster trust and participation among an already burdened population, reinforce the person-centered approach underpinning the co-design process, and distinguish our work from the broader caregiving app literature, in which faith-based leadership has rarely been incorporated into intervention co-design [10]. In addition, we consulted not only target end users but also other key participants, who are often underrepresented in human-centered design studies but can provide further important practical and experiential insights [21]. In our study, for instance, clinicians provided suggestions informed by their experience using the sister app and their clinical work with dementia care partners and persons living with dementia. Finally, although our human-centered design process was prolonged, our robust design process at various time points was a valuable means of implementing and meaningfully designing the app for end users.

Although our second qualitative category, desired features for a dementia caregiving app, identified recommendations such as a Spanish-language version and tailoring the app to accommodate care partners with multiple care recipients, these findings are consistent with prior mHealth design literature and thus offer limited novelty as stand-alone design recommendations [24-26]. In contrast, our primary qualitative contributions lie in identifying facilitators of care partner engagement, which highlights design elements that may promote sustained use of mHealth interventions and ultimately reduce care partner burden. Through our co-design approach, care partners identified features that supported continued engagement with the intervention rather than simply improving usability. For instance, participants emphasized the importance of an integrated journaling feature, describing it as a mechanism to reflect on caregiving experiences and maintain motivation to use the app over time (Figure 3). This aligns with emerging evidence that reflective journaling can enhance engagement with dementia caregiving interventions by encouraging self-monitoring and reinforcing care partner self-efficacy, which may contribute to improved retention [27,28].

In addition to these engagement strategies, the Aliviado Caregiving app integrates a novel machine learning–based decision algorithm into a co-designed intervention that supports care partners in identifying and prioritizing BPSD. The Aliviado Caregiving app holds significant potential to address the existing limitations of mHealth apps designed for care partners of persons living with dementia. Although most dementia care apps offer educational resources or symptom tracking, few offer personalized and tailored guidance to help care partners determine which BPSD should be prioritized for management [10]. By translating complex clinical data into individualized recommendations, we contribute to the advancement of knowledge on mHealth apps by developing and describing how Aliviado Caregiving may offer personalized, contextually relevant learning, recommendations, and support for care partners. Integrating a predictive decision support algorithm within a coproduced mHealth app represents an important step toward more personalized, adaptive dementia caregiving technologies that not only engage care partners but also provide actionable support to reduce caregiving burden.

### Limitations and Future Directions

We aim to establish an effective mHealth app to support marginalized care partners in prioritizing BPSD and accessing evidence-based, nonpharmacologic strategies outside of the health care system to ultimately reduce care partner burden. The next steps include feasibility and acceptability testing of the Aliviado Caregiving app for diverse care partners in a single-arm feasibility study (testing in a real-world environment phase of coproduction), and if successful, this will be followed by a fully powered real-world clinical trial for outcomes (outcomes and impact phase).

Several limitations should be considered. First, representation varied across stages and was constrained by small sample sizes. Rather than aiming to represent all marginalized caregiver populations, this study describes the development of the Aliviado Caregiving app based on feedback from the specific stakeholder groups engaged in the research, while highlighting equity-focused priorities for future testing with more diverse care partner populations. For example, it will be important to obtain the perspectives of Latino care partners to iteratively develop and test a Spanish-language version of the Aliviado Caregiving app, as this population is projected to experience a 7-fold increase in the number of persons living with dementia by 2060 [29]. Second, demographic data collection in stage 2 was optional, which reduced participant burden but limited the interpretability of participant characteristics. Third, all feedback was collected virtually and based on wireframe prototypes and a beta-version app that was not fully functional. Perceptions of usability and user preferences may differ during sustained real-world use. Finally, this design-focused study did not assess the effectiveness of the proposed strategies or the performance of the recommendation component. These outcomes warrant prospective testing, including examination of potential differential performance across care partner subgroups. Furthermore, care partners were not engaged in the co-design process from the absolute inception of the initiative, as the broader Aliviado program, clinical workflows, and infrastructure had already been established, and preliminary app requirements had been identified in a previous design phase with the investigator team and hospice interdisciplinary team members [15,16].

### Conclusions

In this study, we described the iterative, human-centered design process used to develop the Aliviado Caregiving app, providing an example of how common limitations in mHealth app development can be addressed to strengthen the resulting intervention. The long-term objective will be to make the app broadly available to the general public to increase access to high-quality, low-cost BPSD self-management strategies to help achieve health equity and reduce care partner burden, stress, and burnout.

## Appendix

Multimedia Appendix 1 COREQ checklist.

## Abbreviations

BPSD: behavioral and psychological symptoms of dementia
CAB: care partner advisory board
COREQ: Consolidated Criteria for Reporting Qualitative Research
IRB: Institutional Review Board
mHealth: mobile health

## Data Availability

The data supporting the findings of this study are not publicly available due to ethical and privacy considerations, as they contain sensitive information from participants. Access to deidentified data may be granted by the principal investigator upon reasonable request, subject to institutional review board approval and data use agreements.
